# Affibody-mediated PET imaging of HER3 expression in malignant tumours

**DOI:** 10.1038/srep15226

**Published:** 2015-10-19

**Authors:** Maria Rosestedt, Ken G. Andersson, Bogdan Mitran, Vladimir Tolmachev, John Löfblom, Anna Orlova, Stefan Ståhl

**Affiliations:** 1 Preclinical PET Platform, Uppsala University, Uppsala, Sweden; 2 Division of Protein Technology, KTH Royal Institute of Technology, Stockholm, Sweden; 3 Department of Immunology, Genetics and Pathology, Uppsala University, Uppsala, Sweden

## Abstract

Human epidermal growth factor receptor 3 (HER3) is involved in the progression of various cancers and in resistance to therapies targeting the HER family. *In vivo* imaging of HER3 expression would enable patient stratification for anti-HER3 immunotherapy. Key challenges with HER3-targeting are the relatively low expression in HER3-positive tumours and HER3 expression in normal tissues. The use of positron-emission tomography (PET) provides advantages of high resolution, sensitivity and quantification accuracy compared to SPECT. Affibody molecules, imaging probes based on a non-immunoglobulin scaffold, provide high imaging contrast shortly after injection. The aim of this study was to evaluate feasibility of PET imaging of HER3 expression using ^68^Ga-labeled affibody molecules. The anti-HER3 affibody molecule HEHEHE-Z08698-NOTA was successfully labelled with ^68^Ga with high yield, purity and stability. The agent bound specifically to HER3-expressing cancer cells *in vitro* and *in vivo*. At 3 h pi, uptake of ^68^Ga-HEHEHE-Z08698-NOTA was significantly higher in xenografts with high HER3 expression (BT474, BxPC-3) than in xenografts with low HER3 expression (A431). In xenografts with high expression, tumour-to-blood ratios were >20, tumour-to-muscle >15, and tumour-to-bone >7. HER3-positive xenografts were visualised using microPET 3 h pi. In conclusion, PET imaging of HER3 expression is feasible using ^68^Ga-HEHEHE-Z08698-NOTA shortly after administration.

The human epidermal growth factor receptor type 3 (HER3 or human ErbB3) is a transmembrane tyrosine kinase receptor belonging to the HER3 family. This receptor family is involved in regulation of normal cellular functions, such as proliferation, motility and apoptosis suppression. Dysregulation of receptors from the HER family is associated with malignant phenotype^1^. Receptors belonging to the HER-family have an extracellular domain, an intracellular tyrosine kinase domain, which activates downstream signaling pathways, and an intracellular C-terminal tail. HER3, however, has an inactive intracellular tyrosine kinase domain, and its activation depends on a hetero-dimer formation with other HER-family members, preferably HER2^2^. The HER2-HER3 unit activates downstream signalling pathways, such as PI-3K/Akt and MAPK/MEK and is considered as one of the most potent hetero-dimers in tumorigenesis^3^. Strong evidence points to the involvement of HER3 in various cancers, such as breast, prostate and colorectal cancer, and its role in the resistance of these tumours to receptor tyrosine kinase-targeted therapies^4^,^5^. In response to trastuzumab therapy in HER2 overexpressing breast cancer, HER3 can become upregulated and increase the signalling ability of HER2 as a compensation for its inhibition, which cause resistance to therapy^6^,^7^. There are also strong evidence that HER2-HER3 and HER1-HER3 heterodimers are involved in the progression of androgen independent prostate cancer^8^ and promote its invasiveness^9^. It was also demonstrated that autocrine activation of HER2 with heregulin occurs through dimerization with HER3 in colon cancer^10^.

The importance of HER3 in various cancers points to its potential as a molecular target in anti-cancer therapy, which has stimulated the development of appropriate pharmaceuticals^3^,^5^,^11^,^12^. Currently, several HER3-targeting monoclonal antibodies are evaluated in clinical trials^13^,^14^. To make anti-HER3 therapy effective, patients with HER3 overexpressing tumours have to be identified. Currently, tumour biopsy material is predominantly used for molecular profiling, which is associated with several drawbacks. Biopsies are invasive and not easily repeatable, and target expression heterogeneity could lead to false-negative findings^15^. For HER3, biopsies from primary tumours are not informative since HER3 overexpression often occurs in response to therapy. Frequent sampling is therefore required, which is undesirable due to the invasiveness of the procedure.

Radionuclide molecular imaging of therapeutic targets is a non-invasive procedure which can be easily repeated. Imaging-based diagnostics reduces errors occurring due to inter- and intratumoural heterogeneity of expression^16^,^17^. The use of radiolabelled anti-HER3 monoclonal antibodies is a straightforward way to develop imaging agents^18^. However, small peptide-based imaging probes can provide higher specificity and sensitivity than intact monoclonal antibody due to better tissue penetration, faster blood clearance and absence of non-target-specific accumulation in tumours due to the “enhanced permeability and retention effect” (EPR)^16^. During the last decade, it has been demonstrated that high-affinity scaffold proteins are well-suited as imaging probes in oncology^19^. Affibody molecules are small (7 kDa) and robust three-helical scaffold proteins. Due to the very small size, the biodistribution of affibody molecules is characterized by a rapid tumour penetration and fast blood clearance of unbound tracer. This facilitates high-contrast imaging after only a few hours post injection (pi), which has been demonstrated in preclinical and clinical studies^20^,^21^,^22^. The small size of affibody molecules also minimizes the EPR effect that is often an issue with antibody-based tracers. In clinics, the anti-HER2 affibody molecule labelled with indium-111 for SPECT^21^ and gallium-68 for PET^22^ demonstrated ability to discriminate between breast cancer lesions with high and low receptor expression. Using different display techniques, high-affinity binders to different molecular targets can be selected. Recently, affibody molecules with low picomolar affinity to HER3 have also been developed^23^.

The main challenges in imaging of HER3 are a low receptor expression in tumours, usually below 50,000 receptors per cell^24^, together with significant HER3 expression in normal tissues^25^. In order to meet the requirements of high contrast imaging of targets with such low expression, a low picomolar affinity of the targeting molecule is necessary^26^. An affibody molecule targeting HER3 (Z08698) has been engineered to 50 pM affinity for the receptor^23^. Additionally, Z08698 was selected to have cross-reactivity with the murine HER3 counterpart, mErbB3^27^, which is important to make murine models relevant for development and evaluation of imaging properties of the new targeting agent.

Feasibility of *in vivo* imaging of HER3 expression was demonstrated using an anti-HER3 HEHEHE-Z08699 affibody molecule labelled with technetium-99 m (T½ = 6 h) using ^99m^Tc(CO)_3_^27^. Our previous experience with the development of affibody-based imaging agents for targets with endogenous expression in normal tissues (e.g. IGF1R, EGFR) has indicated that imaging contrast could be improved at later time points (i.e. at 24 h pi)^28^,^29^. To test if an extension of the time interval between injection and imaging would increase the imaging contrast, we evaluated anti-HER3 affibody molecules, HEHEHE-Z08698-NOTA and HEHEHE-Z08699-NOTA labelled with long-lived indium-111 (T_½_ = 2.6 d)^30^. Indeed, we have demonstrated that ^111^In-HEHEHE-Z08698-NOTA provides better contrast compared to the ^99n^Tc-labelled tracer^30^. Additionally, we also found that tumour-to-non-tumour ratios, which determine imaging contrast, did not significantly improve after 4 h pi^30^. From this, we concluded that short-lived radionuclides, such as ^18^F and ^68^Ga should be suitable for high contrast HER3 imaging.

Positron emission tomography (PET) provides better resolution, higher sensitivity and superior quantification accuracy than single photon emission computed tomography (SPECT)^31^. Gallium-68 (T½ = 67.7 min) is a short-lived generator-produced positron emitting isotope. Clinical studies have demonstrated that ^68^Ga-labelled anti-HER2 affibody molecules provide best-in-class PET images up to 4 h pi^22^. The NOTA (2,2′,2″-(1,4,7–triazonane–1,4,7–triyl)triacetic acid) chelator permits stable labelling of affibody molecules with ^68^Ga^32^,^33^. However, biodistribution of ^111^In and ^68^Ga-labelled NOTA-conjugated affibody molecules are not identical.

The aim of this study was to test the hypothesis that ^68^Ga-HEHEHE-Z08698-NOTA would enable PET imaging of HER3 expression in tumour xenografts and discriminate between tumours with high and low HER3 expression. Cancer cell lines expressing different levels of HER3; BT474 (breast cancer), BxPC-3 (pancreatic cancer), LS174T (colorectal cancer) and A431 (epidermoid cancer) were hence used in this study.

## Results

### Labelling of HEHEHE-Z08698-NOTA with ^68^Ga

Labelling of HEHEHE-Z08698-NOTA with ^68^Ga at 95 °C provided a labelling yield of 85 ± 5.4% (n = 10) and the purity after size-exclusion purification (NAP-5 column) was >98%. The specific radioactivity was in the range 16–19 GBq/μmol. The conjugate was stable under EDTA-challenge and did not show any release of radioactivity as compared to the control (95 ± 0.9% of radioactivity was associated with protein after EDTA challenge vs 94 ± 0.9%). The conjugate was also stable in an *in vitro* incubation in mouse serum, both mimicking blood concentration after injection of 2 and 70 μg protein dose injections. According to ITLC analyses, 99 ± 0.8% of radioactivity was associated with the protein for the dose of 2 μg; 97 ± 0.2% for the dose of 70 μg, and according to size exclusion analyses −96.6 ± 0.2% and 96.9 ± 0.3%, respectively.

### *In vitro* specificity test and cellular processing of ^68^Ga-HEHEHE-Z08698-NOTA

In an *in vitro* specificity assay, a pre-saturation of receptors with a non-labelled affibody molecule significantly decreased (*n* = 3, *p* values were 0.0001 for BT474, 0.003 for BxPC-3, 0.0007 for LS174T, and 0.0005 for A431 cells) the binding of ^68^Ga-HEHEHE-Z08698-NOTA to HER3-expressing cell lines. This shows that the binding was saturable and suggests that ^68^Ga-HEHEHE-Z08698-NOTA binds specifically to HER3-receptors.

**Figure 1:**
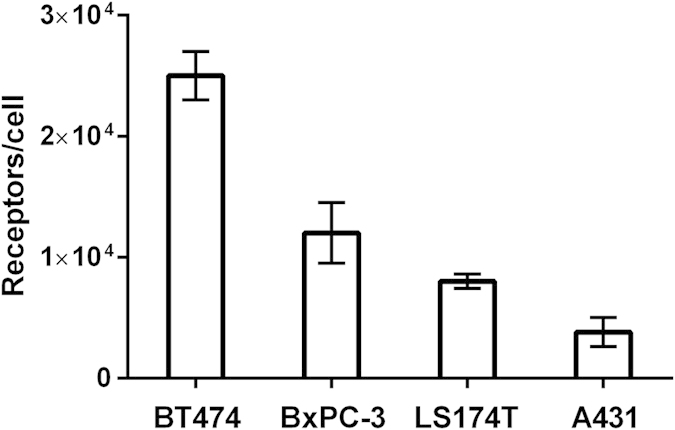
*In vitro* HER3 receptor expression in studied tumour cell lines. Results are presented as an average of 6–9 samples ± SD.

The estimated HER3 expression was 25 ± 2 × 10^3^ for BT474 cells, 12 ± 2 × 10^3^ for BxPC-3 cells, 8.0 ± 0.6 × 10^3^ for LS174T cells and 4 ± 1 × 10^3^ receptors for A431 cells (Fig. 1).

**Figure 2:**
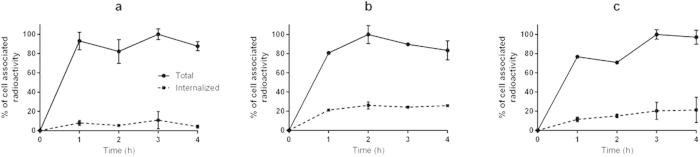
Binding and internalization of ^68^Ga-HEHEHE-Z08698-NOTA by (a) LS174T (b) BxPC-3 and (c) BT474 cells. Cells were continuously incubated with labelled conjugate at 37 °C. Data is presented as mean values from 3 cell dishes ± SD. Error bars might not be seen because they are smaller than the symbols.

The pattern of cellular uptake of ^68^Ga-HEHEHE-Z08698-NOTA during continuous incubation with cells having moderate to high HER3 expression (LS174T, BxPC-3, BT474) was similar among the tested cells (Fig. 2). The maximum cell-bound radioactivity was reached within 2 h and was followed by a plateau. However, the internalization pattern was different. BT474 and BxPC-3 cells demonstrated relatively rapid internalization of radioactivity, 20–30% of cell-associated radioactivity was internalized after 4 h. This was in agreement with data for ^111^In-HEHEHE-Z08698-NOTA^30^. The internalization of radioactivity by LS174T was much slower and did not exceed 5% of cell associated radioactivity at the end of the observation.

### *In vivo* studies

**Figure 3:**
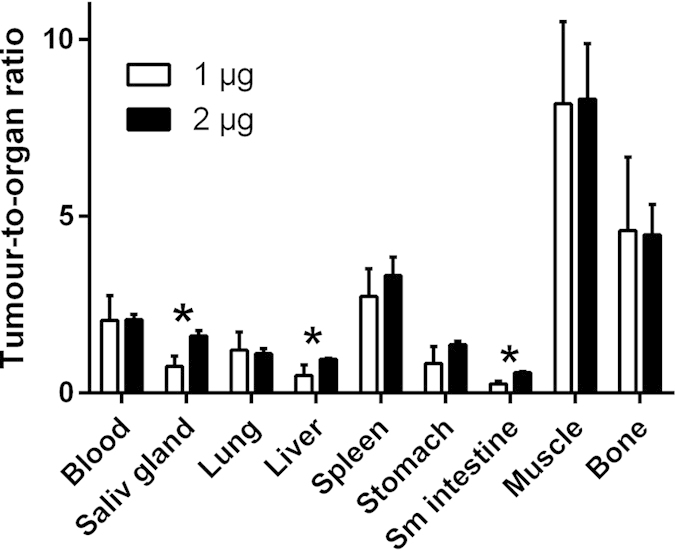
Tumour-to-organ ratios 1 h pi of ^68^Ga-HEHEHE-Z08698-NOTA in LS174T tumour bearing Balb/c nu/nu mice at injected protein dose of 1 and 2 μg. Results are presented as average of 4 animals ± SD. *indicates significant (p < 0.05) difference between values for 1 and 2 μg.

Two groups of mice bearing LS174T xenografts were injected with 1 and 2 μg of ^68^Ga-HEHEHE-Z08698-NOTA and radioactivity distribution was measured at 1 h pi (Table 1). Data for biodistribution in normal tissue of 1 μg ^68^Ga-HEHEHE-Z08698-NOTA were in good agreement with data for indium and technetium labelled variants^27^,^30^. The radioactivity uptake in tumours was not influenced by the injected protein dose. However, the radioactivity uptake in normal mErbB3 expressing tissues significantly decreased when 2 μg of protein was injected (*n* = 4, *p* = 0.02 for liver and small intestine, *p* = 0.001 for salivary glands). Radioactivity uptake in normal organs without mErbB3 expression and radioactivity concentration in blood did not change. As the result, tumour-to-non-tumour ratios were significantly improved for liver (*p* = 0.02), intestines (*p* = 0.003), and salivary glands (*p* = 0.002) (Fig. 3). The stability of the conjugate *in vivo* was analysed 15 min pi in blood serum, and did not show any release of radioactivity (98 ± 1% of radioactivity was associated with the protein according to ITLC analyses and 94 ± 3% according to size exclusion analysis).

Comparison of biodistribution profiles of ^68^Ga-HEHEHE-Z08698-NOTA (2 μg) in mice bearing LS174T xenografts at 1 and 3 h pi showed that the radiolabeled conjugate has rapid blood and whole body clearance (Tables 1 and 2). Radioactivity concentration in blood was already below 1%ID/g by 1 h pi and significantly decreased by 3 h pi (*n* = 4 for 1 h, *n* = 8 for 3 h, *p* = 0.0007). High radioactivity uptake in kidneys indicated that the main excretion pathway was renal with a subsequent reabsorption.

**Figure 4:**
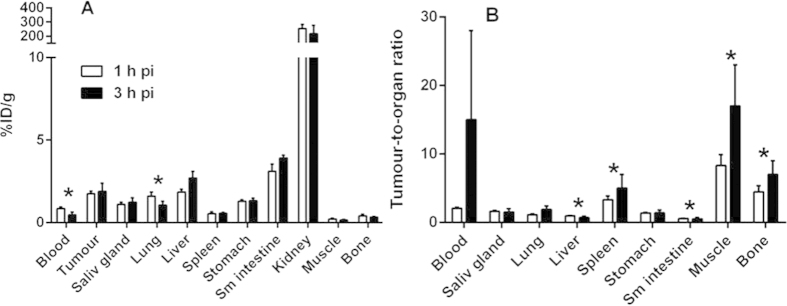
(**A**) Biodistribution and (**B**) tumour-to-organ ratios at 1 and 3 h pi of 2 μg of ^68^Ga-HEHEHE-Z08698-NOTA in LS174T tumour bearing Balb/c nu/nu mice. Results are presented as average of 4–8 animals ± SD. *indicates significant (p <`0.05) difference between values at 1 and 3 h pi.

Radioactivity uptake in tumour was already higher than radioactivity in blood by 1 h pi (Fig. 4A). The tumour uptake at 3 h pi remained at the same level as 1 h pi, but clearance from normal tissues resulted in significant increase of tumour-to-spleen (*p* = 0.009), tumour-to-intestines (*p* = 10^−8^), tumour-to-bone (*p* = 0.002), and tumour-to-muscle ratios (*p* = 0.001) (Fig. 4B). However, radioactivity uptake in tumours was lower than the uptake in liver and small intestine (mErbB3-expressing organs) by 3 h pi. Further biodistribution experiments were performed at 3 h pi using injected dose of 2 μg.

**Figure 5:**
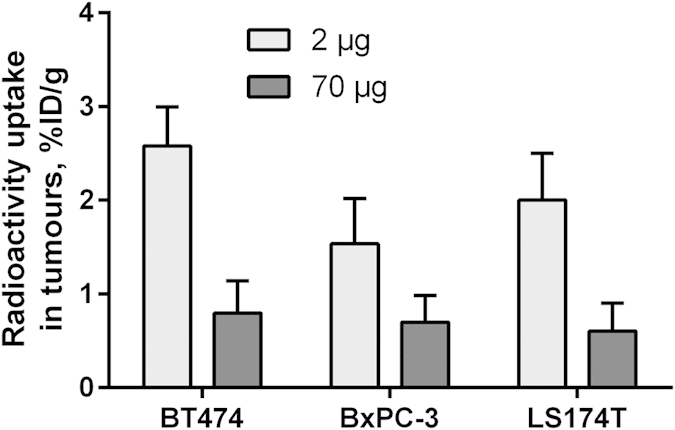
*In vivo* binding specificity of ^68^Ga-HEHEHE-Z08698-NOTA to HER3 expressing xenografts at 3 h pi. Results are presented as average of 4–8 animals ± SD.

**Figure 6:**
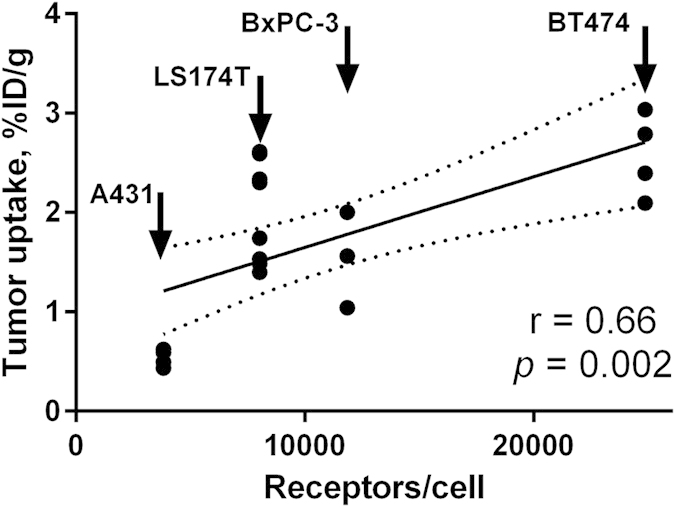
Correlation of tumor uptake with HER3 expression level in respective cell line at 3 h pi of ^68^Ga-HEHEHE-Z08698-NOTA. Linear regression analysis (GraphPad Prism, version 6 for Windows GraphPad Software) was used for data treatment. Dotted lines represent 95% confidence interval. Deviation from linearity was non-significant (*p* = 0.3) according to runs test analysis.

The results from the *in vivo* specificity assay (Fig. 5 and Table 2) demonstrated saturable uptake of ^68^Ga-HEHEHE-Z08698-NOTA in xenografts with high HER3 expression (BT474, BxPC-3 and LS174T). Moreover, injection of a saturating peptide dose caused substantial decrease in uptake in mErbB3 expressing tissues, i.e. salivary gland, lung, liver, stomach and small intestines (Table 2), hence demonstrating HER3/mErbB3-specific uptake of ^68^Ga-HEHEHE-Z08698-NOTA *in vivo*. Uptake in BT474, BxPC-3 and LS174T xenografts with high HER3 expression was significantly higher than uptake in A431 xenografts with low expression (*p*-values were 0.002, 0.001, 0.0002, respectively) (Table 2). Radioactivity uptake in tumours correlated significantly (p < 0.05) with receptor expression in cell lines (Fig. 6). The biodistribution profile in normal tissues was reproducible to some extent, with some variations that could partially be attributed to the xenograft models (e.g. BT474 xenografts require estradiol pellet implantation) and partially to batch-to-batch variability of mice. In xenografts with high HER3 expression, tumour-to-blood ratios were over 20, tumour-to-muscle over 15, and tumour-to-bone over 7.

### Imaging studies

**Figure 7:**
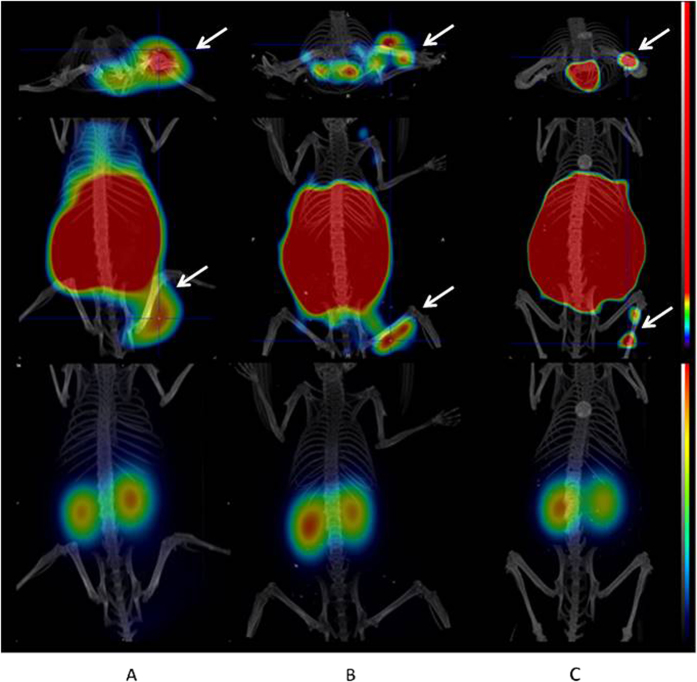
microPET/CT images of mice bearing (A) BT474, (B) BxPC-3 and (C) LS174T xenografts at 3 h pi of 2 μg/0.7 MBq of ^68^Ga-HEHEHE-Z08698-NOTA. The transaxial (upper row) and coronal (middle row) images have a scale 0–3%ID/g, coronal image (low row) - 0–100%ID/g. Arrows indicate tumours.

Images acquired 3 h pi with ^68^Ga-HEHEHE-Z08698-NOTA for mice bearing BT474, BxPC-3 and LS174T xenografts, are presented in Fig. 7. As expected, the highest accumulation of radioactivity was observed in the kidneys, which exceed the uptake in any other organ or tissue. Organs with a normal expression of mErbB3 (liver and gastro-intestinal tract) also demonstrated a radioactivity uptake. All tumours were clearly visualized. Images confirmed the rapid blood clearance of the radiolabeled conjugate, shown by the low background radioactivity.

## Discussion

Earlier, radiolabelled anti-HER3 antibodies, their fragments, and a natural ligand, heregulin, were proposed for HER3 imaging using SPECT and PET modalities^18^,^34^,^35^. Direct comparison between affibody molecules and antibodies have demonstrated that affibody molecules provide higher tumour-to-organ rations than antibodies^36^,^37^ and therefore higher imaging contrast. In the case of molecular targets with low expression in normal tissues, such as HER2^36^,^37^, CAIX^38^ or PDGFRb^39^, a high contrast was obtained within a few hours after injection because of rapid clearance of non-bound tracer. However, a high contrast was obtained only by 8 to 24 h pi, when molecular targets with moderate to high expression in normal tissues, e.g. EGFR^29^ or IGF-1R^40^, were visualized using affibody molecules. It has to be noted that the internalization of affibody molecules after binding to molecular targets is usually rather slow, 20% per day or less^28^,^29^,^41^. The majority of affibody molecules remain membrane bound in normal tissues. A clearance of the tracer from blood shifts the binding equilibrium and results in release of affibody molecules from tissues to blood. HER3/ErbB3 has ample expression in normal tissues^25^. Therefore, an initial characterisation of affibody molecules was performed with relatively long-lived nuclides, ^99m^Tc (T_½_ = 6 h) and ^111^In (T_½_ = 2.6 d)^27^,^30^. Surprisingly, the blood clearance was faster than we expected. For example, the tumour-to-blood ratio for BT474 xenografts at 4 h pi was 7 ± 3 and 12 ± 3 for ^99n^Tc(CO)_3_-HEHEHE-Z08699 and ^111^In-HEHEHE-Z08698-NOTA. A possible explanation for such rapid blood clearance might be faster internalization of anti-HER3 affibody molecules (Fig. 2). The internalized fraction of ^68^Ga-HEHEHE-Z08698-NOTA at 4 h (approximately 20%) is the same as for other affibody molecules at 24 h^28^,^29^,^41^.

This finding suggested that positron-emitting radionuclide gallium-68 with a half-life of 67.6 minutes can be used for imaging of HER3. The use of PET as imaging modality could contribute to higher accuracy for molecular diagnostics and quantitative information of the molecular target expression^31^. ^68^Ga is produced by a generator from a long-lived ^68^Ge (T_½_ = 271 d), and cyclotrons are hence not needed. Additionally, the use of short-lived ^68^Ga will reduce the radiation dose burden to patients.

Due to the short half-live of ^68^Ga, HEHEHE-Z08698-NOTA was labelled at 95 °C to facilitate incorporation of the metal in the chelator. These conditions provided high labelling yield, and the Ga-NOTA complex was stable under EDTA challenge. The low level of radioactivity uptake in bones and rapid blood clearance confirmed high stability of the gallium-NOTA complex in the *in vivo* experiments. However, elevated temperatures as well as the acidic pH during labelling might cause denaturation and potentially destroy the binding capacity of the proteins. Binding of ^68^Ga-HEHEHE-Z08698-NOTA to HER3 expressing cells was specific, which demonstrated preserved binding capacity of the conjugate. This was in agreement with previous data on high thermal and chemical stability of the affibody scaffold^20^. The radiolabeled conjugate demonstrated rapid binding to cells *in vitro* as well as a relatively rapid internalization that was somewhat slower for cells with a moderate HER3 expression (LS174T).

A challenge with targeting of HER3 is not only its low expression in tumour, but also the expression in normal tissues. Particularly challenging is expression in liver, which is a large and well perfused organ and has a well-fenestrated vasculature. This could potentially cause poor contrast of HER3-expressing lesions in liver and also the reduction of the tracer bioavailability by entrapment in the liver. Earlier experiments were performed with an injected affibody dose of 1 μg^27^,^30^. We hypothesized that by a small increase of the injected protein dose, we could partially saturate the receptors in the liver, but hopefully not in the xenografts. A biodistribution of ^68^Ga-HEHEHE-Z08698-NOTA in mice bearing LS174T xenografts was compared at injected doses of 1 and 2 μg (Table 1, Fig. 3). At a higher injected dose, the radioactivity uptake in normal organs with mErbB3 expression (salivary glands, liver, stomach and small intestines) decreased approximately two-fold, whereas the radioactivity concentration in blood and tumour remained at the same levels. These findings demonstrated that even small differences in protein dosage could lead to changes in imaging contrast, and in a clinical setting the optimization of injected protein should be given high priority. We did not increase the injected dose further, as the results of the previous study with anti-HER2 affibody molecules have shown that increase of affibody dose to 5 μg per mouse causes appreciable reduction of tumour uptake in LS174T xenografts expressing 4 × 10^4^ receptors per cell^42^. There was no difference in the uptake in LS174T xenografts between 1 and 3 h pi, but clearance of radioactivity from normal tissues resulted in significant increase of tumour-to-spleen, tumour-to-bone, tumour-to-intestine and tumour-to-muscle ratios (Fig. 4B). The 2 μg dose was chosen for further experiments and injected into mice bearing high (BT474 and BxPC-3) and low (A431) HER3 expressing xenografts.

*In vivo* binding specificity was tested by a saturation assay, by comparison of biodistribution after injection of 2 and 70 μg of ^68^Ga-HEHEHE-Z08698-NOTA. Uptake in xenografts with high HER3 expression (BT474, BxPC-3 and LS174T) was significantly lower (Fig. 5), which demonstrated saturable binding, thus suggesting HER3-specificity. Cross-reactivity of anti-HER3 affibody molecule to mouse counterpart of HER3 (mErbB3) was demonstrated by saturable binding to mErbB3-expressing murine organs: salivary glands, lung, liver, stomach and small intestines (Table 2). The cross-reactivity of ^68^Ga-HEHEHE-Z08698-NOTA to mErbB3 allowed us to model all interactions of the traces in murine model, which is essential for further clinical translation. Importantly, the tumour uptake correlated well with the HER3 expression in cell lines (*p* = 0.002, r = 0.66) (Fig. 6). Furthermore, the uptake of ^68^Ga-HEHEHE-Z08698-NOTA in xenografts with high expression (BT474, BxPC-3, and LS174T) was considerable higher than uptake in A431 xenografts with low HER3 expression. In the case of clinical translation, this might provide a straightforward way to discriminate between metastases with high and low expression.

In all models, the blood clearance was very fast, which resulted in high tumour-to-blood ratios already at 3 h pi. This is essential, as blood-born radioactivity contributes to radioactivity content in all organs and determines contrast of imaging *in vivo*. Interestingly, ^68^Ga-HEHEHE-Z08698-NOTA provided higher tumour-to-blood ratios at 3 h pi in comparison with ^99m^Tc-HEHEHE-Z08699 and ^111^In-HEHEHE-Z08698-NOTA at 4 h pi^27^,^30^. This might be partially explained by a more optimal level of the injected dose. Another factor contributing to the difference might be influence of the radionuclide-chelator complex. Complexes of indium and gallium with NOTA both have trigonal prismatic geometry^43^,^44^, but the prismatic geometry is appreciably distorted in the case of bulky indium. Conceivably, difference in the complex geometry may influence both on-target and off-target interaction of labelled peptides. This was documented earlier for both short peptides^45^,^46^ and affibody molecules^32^,^33^. Whatever the reason is, ^68^Ga-HEHEHE-Z08698-NOTA provides the most favourable biodistribution properties among HER3-targeting affibody-based imaging probes. The use of PET would further enhance the sensitivity of imaging HER3 expression. Only a few other agents for HER3-imaging have been reported. Quantitative *in vivo* data have been provided only for ^89^Zr-labelled anti-HER3 antibody RG7116^18^ and an ^111^In-labelled natural ligand of HER3, heregulin-β1 (HRG), ^111^In-DTPA-HRG^34^. Still, the comparison is complicated, as different cell lines were used for HER3-expressing xenografts, and the expression level has not been quantified by other authors. Besides, *in vivo* saturation data suggest that the both the other agents did not have cross-reactivity with murine ErbB3, and models did therefore not reflect all interactions of the agents. However, the tumour-to-blood ratios for ^68^Ga-HEHEHE-Z08698-NOTA were higher than for ^89^Zr-RG7116 (below 8 by 144 h pi) and ^111^In-DTPA-HRG (7 by 48 h pi).

MicroPET images of all three xenograft models were in good agreement with the biodistribution data. High radioactivity uptake was visualized in kidneys, the excretory organ, as well as in liver and organs with endogenous receptor expression. Nevertheless, tumours were clearly visualized with high contrast to background tissues. Of course, negative contrast in liver (tumour-to-liver ratios below 1) is a drawback to the imaging agent in oncology. However, it should to be noticed that anti-HER3 imaging agent would not be used for finding of lesions, but rather as a secondary agent that would give information of target expression in already identified lesions.

The high renal uptake of ^68^Ga-HEHEHE-Z08698-NOTA is an apparent issue of this tracer. Peptides with molecular weight of less than 12 kDa are filtered freely through glomerular membrane and undergo a reabsorption in proximal tubuli^47^,^48^. In the case of residualizing radiometal labels, the reabsorption is followed by a prolonged retention of radionuclides in kidneys^48^. There are several clinical methods that allow the inhibition of tubular reabsorption of the radiopeptides during the peptide receptor radionuclide therapy, such as co-administration of basic amino acids, the plasma expander Gelofusine or albumin fragments^47^,^48^. High level of renal radioactivity uptake is a common feature of affibody molecules^20^,^29^,^39^,^40^. Commonly used methods for reduction of renal reabsorption, such as the use of cationic amino acids or Gelofusine have a minor effect if any^49^. However, anti-HER2 affibody molecule labeled with indium-111 visualized a small adrenal metastasis^21^ although it has similar renal uptake in murine models as uptake of ^68^Ga-HEHEHE-Z08698-NOTA. This indicates that ^68^Ga-HEHEHE-Z08698-NOTA might be used for imaging of HER3 expression of metastases located in lumbar area.

In conclusion, this study presented ^68^Ga-HEHEHE-Z08698-NOTA as a promising agent for PET-imaging of malignant HER3 expressing tumours, already providing a good imaging contrast by 3 h pi and enabled discrimination between xenografts with high and low HER3 expression. However, the high renal accumulation as well as the high liver uptake due to endogenous HER3 expression may be a limiting factors for visualization of abdominal tumours using this agent.

## Materials and Methods

### Materials

The anti-HER3 affibody molecule HEHEHE-Z08698-NOTA was produced and purified as described previously^30^. Cells used during *in vitro* and *in vivo* experiments were purchased from American Type Tissue Culture Collection (ATCC via LGC Promochem, Borås, Sweden). The cell lines A431 (epidermoid carcinoma), LS174T (colorectal carcinoma), BxPC-3 (pancreatic carcinoma) and BT474 (breast carcinoma), were cultured in RPMI media supplemented with 10% fetal bovine serum (FBS) and 1% Penicillin/Streptomycin (PEST) and grown in incubator at 37°C and 5% CO_2_. Trypsin-EDTA (0.25% trypsin, 0.02% EDTA in buffer, Biochrom AG, Berlin Germany) was used to detach cells. Germanium-68/gallium-68 generator (Eckert & Ziegler, Eurotope GmbH, Berlin) was eluted with 0.1M HCl. Buffers for labelling were purified from metal contamination by Chelex 100 resin (Bio-Rad Laboratories). The purity of labelled Affibody molecule was verified by radio instant thin-layer chromatography (ITLC, 150-771 DARK GREEN, Tec-Control Chromatogrphy strips from Biodex Medical Systems, New York, USA) and cross-validated by sodium dodecyl sulfate polyacrylamide gel electrophoresis (SDS-PAGE). The distribution of radioactivity along the chromatography strips and SDS gels was measured by a Cyclone^TM^ Storage Phosphor System using the image analysis software OptiQuant^TM^ (PerkinElmer). Radioactivity content in samples was measured using automated gamma-counter with 3-inch NaI(Tl) detector (1480 WIZARD, Wallac Oy).

Data were assessed by an unpaired, two-tailed t-test using GraphPad Prism (version 6 for Windows GraphPad Software) in order to determine significant differences (p < 0.05). Obtained values are presented as average with standard deviation if not stated otherwise.

### Labelling of HEHEHE-Z08698-NOTA with ^68^Ga

HEHEHE-Z08698-NOTA (50 μg in 9.3 μl PBS) was mixed with 200 μl ^68^Ga-chloride solution (100–120 MBq) and 300 μL ascorbic acid buffer, 1 M, pH 3.6, was added for pH adjustments. The mixture was incubated for 15 min at 95 °C, no attempts to label affibody conjugate at room temperature were done. A small aliquot of 1 μl was taken and analysed by ITLC eluted by 0.2 citric acid, pH 2.0. Radiolabeled affibody molecules remain at the application point while free ^68^Ga migrates with the solvent front (R_*f*_ = 1). A blank experiment not including an affibody molecule was performed to validate the analytical system, and it showed that less than 1.3% of radioactivity remained at the application point (R_*f*_ = 0). ITLC analytical system was also cross-validated by SDS-PAGE analysis (200 V, NuPAGE 4–16% Bis-Tris Gel, Invitrogen AB, Carlsbad, USA). NAP-5 columns (GE Healthcare, USA) were used for purification of conjugate to ensure high radiochemical purity. For stability testing, two samples of the conjugate were diluted with 500-fold molar excess of EDTA in PBS, and two control samples were diluted with equal amount of PBS and incubated at room temperature. After 1 h incubation, samples were analysed by ITLC. Additionally, stability of the conjugate was analysed in mouse serum *in vitro*. The conjugate was incubated for 15 min at 37 °C in the serum in concentrations corresponding to the concentrations after injection of 2 and 70 μg. Samples were further analysed by ITLC and size exclusion NAP-5 columns.

### *In vitro* specificity test and cellular processing of ^68^Ga-HEHEHE-Z08698-NOTA

The receptor binding specificity of ^68^Ga-HEHEHE-Z08698-NOTA was studied using the BT474, BxPC-3, LS174T and A431 cell lines. The cellular processing was studied using cell lines with high receptor density, BT474, BxPC-3 and LS174T.

*In vitro* specificity test was performed in triplicates according to the method described earlier^41^. Briefly, a solution of ^68^Ga-HEHEHE-Z08698-NOTA (0.1 nM) was added to six cell-containing Petri dishes. To saturate the receptors, a non-labelled affibody molecule (500 nM) was added to three control dishes 15 min before adding the radiolabeled conjugate. Cells were incubated at 37 °C for 1 h, the media was collected and the cells were detached by trypsin-EDTA solution and collected. The radioactivity in media and cells was measured to calculate the fraction of cell-bound radioactivity.

Measurements of total uptake and internalization rate in cellular processing experiment was performed according to the method previously described^41^. Cells were incubated with labelled conjugate (0.1 nM) at 37 °C. At 1, 2, 3 and 4 h after incubation start, media from a set of three dishes was removed, and the cells were treated with 0.2 M glycine buffer containing 4 M urea, pH 2.5, for 5 minutes on ice, in order to collect the membrane-bound radioactivity. To collect internalized radioactivity, the cells were treated with 1 M NaOH and incubated at 37 °C for 30 min. The percentage of internalized radioactivity was calculated for each time point.

The receptor density was estimated for all studied cell lines according to a previously described method^28^.

### *In vivo* studies

All animal experiments were planned and performed in accordance with national legislation on laboratory animals’ protection and were approved by the local Ethics Committee for Animal Research in Uppsala.

To evaluate the targeting ability and biodistribution of radiolabeled Affibody molecule, female BALB/C nu/nu mice bearing BT474, BxPC-3, LS174T and A431 xenografts were used. For implantation, 10 × 10^6^ BT474, 5 × 10^6^ BxPC-3, 2 × 10^6^ LS174T and 10 × 10^6^ A431 cells per mouse were used. BT474 cells were implanted in 50% Matrigel in mice pre-implanted with 17β-estradiol pellets (0.025 mg/d, 20 days, Innovative Research of America, Sarasota, USA). At the time of experiment, the animal weight was in the range of 18.9 ± 1.7 g.

Mice (4–8 per group) were injected iv with (1 MBq) ^68^Ga-HEHEHE-Z08698-NOTA in 100 μl PBS. Protein dose was adjusted to designed amount using non-labelled conjugate. Mice were sacrificed at pre-determined time points by injection of a lethal dose of anaesthesia (20 μl of Ketalar-Rompun per gram body weight; Ketalar (50 mg/ml, Pfizer), 10 mg/ml; Rompun (20 mg/ml, Bayer)) followed by heart puncture and exsanguination with a heparinized syringe. Samples of blood, organs and tumours were collected and uptake of radioactivity in tissues was measured. Tissue uptake was calculated as the percentage of injected radioactivity per gram tissue (%ID/g). Radioactivity uptake in the carcass and gastrointestinal tract was calculated as %ID per whole sample.

Mice with LS174T xenografts were injected with 1 (8 GBq/μmol) or 2 (4 GBq/μmol) μg of affibody molecule and sacrificed at 1 h pi with the aim to defined optimal injected protein dose. One more group injected with 2 μg was sacrificed at 3 h pi with the aim to study biodistribution of labelled conjugate over time. Mice with BT474, BxPC-3 and A431 xenografts were injected with 2 μg of labelled conjugate and sacrificed at 3 h pi.

In order to evaluate if the uptake of ^68^Ga-HEHEHE-Z08698-NOTA in xenografts and ErbB3-expressing organs (lung, liver, stomach, small intestines, salivary gland) was receptor mediated, an additional experiment was performed. ^68^Ga-HEHEHE-Z08698-NOTA was injected iv with injected dose of protein adjusted to 70 μg per mouse by dilution with non-labelled affibody molecule in BT474, BxPC-3 and LS174T xenografted mice. Biodistribution was performed at 3 h pi as described above. The stability of the conjugate in blood was tested *in vivo*. NMRI mice were iv injected with 2 μg of the conjugate, sacrificed 15 min pi and the blood was collected and centrifuged. The serum-supernatant was analysed by ITLC and size exclusion NAP-5 columns.

### Imaging studies

Mice with BT474, BxPC-3 and LS174T xenografts were administered iv with 0.7 MBq of ^68^Ga-HEHEHE-Z08698-NOTA (2 μg, 100 μl PBS) as a single bolus injection via the tail vein. The animals were euthanized 3 h pi and the urinary bladders were excised post-mortem to improve image quality. Each subject was placed in the gantry of the small animal PET/CT scanner (Triumph™ Trimodality System, TriFoil Imaging, Inc., Northridge, CA, USA) and examined by whole body PET acquisition for 30 min in list mode followed by a CT acquisition for 3 min (Field of View (FOV) = 8.0 cm). The PET data were reconstructed into a static image using an ordered subset expectation maximization (OSEM) 3-D algorithm (20 iterations; 8 subsets). The CT raw files were reconstructed using Filter Back Projection (FBP). PET data were reconstructed for attenuation and scatter corrections with their respective CT data. PET and CT images were analysed using PMOD v3.508 (PMOD Technologies Ltd, Zürich, Switzerland).

## Additional Information

**How to cite this article**: Rosestedt, M. *et al.* Affibody-mediated PET imaging of HER3 expression in malignant tumours. *Sci. Rep.* **5**, 15226; doi: 10.1038/srep15226 (2015).

## Figures and Tables

**Table 1:** Biodistribution of ^68^Ga-HEHEHE-Z08698-NOTA in LS174T xenografted mice 1 h pi.

	1 μg	2 μg
Blood	0.9 ± 0.2	0.9 ± 0.1
Tumour	1.7 ± 0.5	1.8 ± 0.1
Salivary glands	2.4 ± 0.4	1.1 ± 0.1*
Lung	1.6 ± 0.7	1.6 ± 0.2
Liver	4 ± 1	1.9 ± 0.2*
Spleen	0.6 ± 0.2	0.5 ± 0.1
Stomach	2.3 ± 0.8	1.3 ± 0.1
Small intestine	7.0 ± 2	3.1 ± 0.4*
Kidney	208 ± 56	255 ± 27
Muscle	0.2 ± 0.1	0.20 ± 0.05
Bone	0.4 ± 0.1	0.4 ± 0.1

Results are presented as percentage of injected dose of radioactivity per gram of tissue (%ID/g). Values are presented as average of 4 animals ± SD.

^*^Uptake was significantly lower (*p* < 0.05).

**Table 2:** Biodistribution of ^68^Ga-HEHEHE-Z08698-NOTA in xenografted mice 3 h pi.

	BT474		BxPC-3		LS174T		A431
	2 μg	70 μg	2 μg	70 μg	2 μg	70 μg	2 μg
Biodistribution, % ID/g							
Blood	0.11 ± 0.02	0.07 ± 0.01^a^	0.068 ± 0.004	0.05 ± 0.01^a^	0.3 ± 0.2	0.08 ± 0.01	0.08 ± 0.02
Tumour	2.6 ± 0.4^b^	0.8 ± 0.3^a^	1.5 ± 0.5^b^	0.7 ± 0.3	2.0 ± 0.5^b^	0.6 ± 0.3^a^	0.54 ± 0.09
Salivary glands	1.8 ± 0.1	0.4 ± 0.1^a^	0.9 ± 0.1		1.4 ± 0.3	0.48 ± 0.06^a^	1.7 ± 0.3
Lung	1.3 ± 0.4	0.42 ± 0.04^a^	0.8 ± 0.1		1.1 ± 0.2	0.38 ± 0.06^a^	1.3 ± 0.2
Liver	4.5 ± 0.7	1.0 ± 0.1^b^	2.5 ± 0.2	0.6 ± 0.1^a^	2.9 ± 0.3	0.63 ± 0.07^a^	3.7 ± 0.9
Spleen	0.6 ± 0.2	0.61 ± 0.06	0.29 ± 0.05		0.5 ± 0.1	0.34 ± 0.1	0.6 ± 0.2
Stomach	1.9 ± 0.4	0.44 ± 0.02^a^	2 ± 1		1.4 ± 0.2	0.5 ± 0.1^a^	1.6 ± 0.4
Small intestine	7 ± 2	0.6 ± 0.1^a^	3.8 ± 0.1		4.5 ± 0.9	0.48 ± 0.06^a^	5.0 ± 0.8
Kidney	189 ± 31	221 ± 16	221 ± 2	226 ± 19	193 ± 52	177 ± 15	196 ± 47
Muscle	0.14 ± 0.03	0.16 ± 0.01	0.11 ± 0.04		0.13 ± 0.03	0.15 ± 0.03	0.17 ± 0.06
Bone	0.3 ± 0.1	0.4 ± 0.1	0.23 ± 0.04		0.29 ± 0.06	0.23 ± 0.04	0.2 ± 0.2
Tumour-to-organ ratio							
Blood	25 ± 6		23 ± 7		15 ± 13		7 ± 1
Muscle	19 ± 5		16 ± 8		16 ± 8		3.2 ± 0.7
Bone	9 ± 4		7 ± 4		7 ± 4		3 ± 2

Values are presented as average of 4 to 8 animals ± SD.

^a^significant (*p* < 0.05) difference between uptake values at injected dose of 2 and 70 μg.

^b^significant (*p* < 0.05) difference with uptake in A431 xenograft.
